# Garlic and Its Bioactive Compounds: Implications for Methane Emissions and Ruminant Nutrition

**DOI:** 10.3390/ani12212998

**Published:** 2022-10-31

**Authors:** Nurul Fitri Sari, Partha Ray, Caroline Rymer, Kirsty E. Kliem, Sokratis Stergiadis

**Affiliations:** 1 Department of Animal Sciences, School of Agriculture, Policy and Development, University of Reading, Reading RG6 6EU, UK; 2 Research Center for Applied Zoology, National Research and Innovation Agency (BRIN), Cibinong 16911, West Java, Indonesia; 3 The Nature Conservancy, Arlington, VA 22203, USA

**Keywords:** garlic, greenhouse gas, ruminant, organosulphur, plant-derived bioactive compounds

## Abstract

**Simple Summary:**

Methane (CH_4_) produced by ruminants contributes as a source of anthropogenic greenhouse gases (GHG). Plant-derived bioactive compounds have been investigated for their potential to reduce CH_4_ emissions from ruminant livestock. Garlic contains bioactive organosulphur compounds, which have been reported to be effective in reducing CH_4_ emissions, but they have demonstrated inconsistent effects in reducing CH_4_ production in the rumen. This might be because different types of garlic-based supplements vary in their concentrations of bioactive compounds. Therefore, further investigation is needed, such as the mode of action and persistence of the bioactive compound, to determine whether these compounds can be used successfully to inhibit rumen methanogenesis. The present review discusses garlic and its potential contribution to reducing CH_4_ production by ruminant animals and discusses how differences in the diet and the concentration of bioactive compounds in garlic might contribute to inconsistent CH_4_ mitigation potential of garlic.

**Abstract:**

Methane (CH_4_) emission from enteric fermentation of ruminant livestock is a source of greenhouse gases (GHG) and has become a significant concern for global warming. Enteric methane emission is also associated with poor feed efficiency. Therefore, research has focused on identifying dietary mitigation strategies to decrease CH_4_ emissions from ruminants. In recent years, plant-derived bioactive compounds have been investigated for their potential to reduce CH_4_ emissions from ruminant livestock. The organosulphur compounds of garlic have been observed to decrease CH_4_ emission and increase propionate concentration in anaerobic fermentations (in vitro) and in the rumen (in vivo). However, the mode of action of CH_4_ reduction is not completely clear, and the response in vivo is inconsistent. It might be affected by variations in the concentration and effect of individual substances in garlic. The composition of the diet that is being fed to the animal may also contribute to these differences. This review provides a summary of the effect of garlic and its bioactive compounds on CH_4_ emissions by ruminants. Additionally, this review aims to provide insight into garlic and its bioactive compounds in terms of enteric CH_4_ mitigation efficacy, consistency in afficacy, possible mode of action, and safety deriving data from both in vivo and in vitro studies.

## 1. Greenhouse Gas Emissions from Ruminants

### 1.1. Greenhouse Gas Emissions from Ruminants and the Contribution of Methane

Ruminants play essential roles in sustainable agriculture, among which is the conversion of renewable resources (grassland, natural pasture, crop residues, or other co-products) into edible food for humans [1]. Worldwide demand for meat and milk is projected to grow by 73 and 58%, respectively, in 2050, compared to 2010, due to continued world population expansion, the emergence of the middle class, increasing incomes, and urbanisation with more emphasis on the developing countries [1,2,3]. Ruminant production needs to provide high-quality food to meet the increasing demands of a growing global population, which can adapt to climate changes and, at the same time, decrease the negative impact on the environment, such as methane (CH_4_), nitrous oxide (N_2_O) and carbon dioxide (CO_2_) emissions and avoid changes in land use such as forest conversion to pasture.

**Figure 1:**
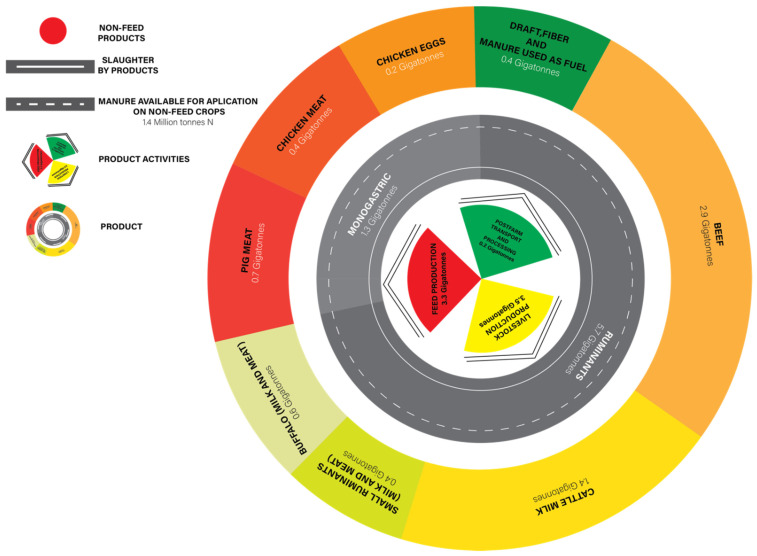
Global livestock emissions from supply chains, production activities, and products (adapted from [1]). This figure is excluded from the CC BY licence under which this article is published.

The livestock sector plays a vital role in climate change, with greenhouse gas (GHG) emissions along livestock supply chains producing seven gigatonnes of CO_2_ equivalents per annum, equalling 14.5% of all human-induced GHG emissions [1,4]. Ruminant production systems are a source of GHG from various activities in the supply chain (Figure 1). Microbial fermentation of feed in the gastrointestinal tract, known as enteric fermentation, is the primary source of CH_4_ emissions from ruminants. Enteric fermentation is the main agricultural source of CH_4_, comprising 39% from dairy, 38% from beef, and 23% from sheep, with emissions from slurry stores and livestock manure handling and spreading accounting for most of the remaining 15%. It is the third largest contributor of GHG after energy and industry [1]. In addition, enteric fermentation in ruminants is the largest source of anthropogenic CH_4_ emissions, contributing between 20 and 25% [5]. Methane emissions from ruminants, in particular, have been a global discussion topic as the global warming potential of CH_4_ is 28 times greater than CO_2_ [6,7,8]. Ruminants also produce large amounts of CO_2_, with a 4:1 CH_4_ to CO_2_ ratio, contributing to ruminants’ total contribution of 8% to anthropogenic GHG emissions [9].

### 1.2. Global Targets for the Mitigation of CH_4_ Emissions

Greenhouse gas emissions must be decreased by 80–90% compared with the emissions in 1990 in developed countries by 2050, according to the European Council Directorate-General for Climate Action [10]. However, agricultural CH_4_ emissions are projected to increase by about 30% by 2050 compared to 2010 under FAOSTAT policies, with a range of 20 to 50% in the integrated assessment model (IAMs) [11,12]. At the same time, the planet will need 70% more food by 2050, and it is predicted that this dramatic increase in production will also cause a 30–40% rise in agricultural emissions due to the growth of the human population and rise in income driving an increased demand for animal protein [13,14,15]. Therefore, food production systems are under pressure to meet these food demands, and climate-smart, sustainable, and environmentally friendly production practices are essential. The various sectors are also challenged with the need of developing more resilient food supply chains under changing climatic conditions while providing safe, affordable, and nutritious foods. Therefore, innovative solutions in climate action and the implementation of appropriate enteric CH_4_ mitigation strategies are required for sustainable food production from ruminants [16].

Global agricultural CH_4_ emissions need to decrease by 24–47% (interquartile range), and CO_2_ emissions need to reach net-zero by mid-century to limit global warming to 1.5 °C [13]. More than 100 countries have recently set targets within the agriculture sector as part of national climate mitigation strategies and commitments. However, only a few (including industrialised countries) have specific targets or are currently designing policies to promote absolute reductions in the agricultural CH_4_ emissions in all sectors [17]. Consequently, policy efforts will need to intensify for the agriculture sector to contribute effectively to limiting the global temperature increase to 1.5 °C, the ambitious end of the Paris Agreement temperature goals [18].

A further challenge in mitigating GHG from the agriculture sector is the rising demand for milk and meat [2,19,20]. While a number of technical solutions are available (such as feed quality, animal health, animal production, and herd management), adoption of these interventions might be hindered by high investment cost in infrastructure and strategies for precision nutrition [1,15,16]. This latter point is critical because there are limited incentives for adopting GHG mitigation technologies under the current emission trading schemes in developed countries; therefore, supportive policies from multi-stakeholders, such as adequate institutional and pro-active governance, are needed to fulfil the sector’s mitigation potential [1,16,19]. This means decreases in GHG emissions need to be viewed holistically, and emissions trade-offs across every stage of different supply chains should be considered for policy-making around GHG mitigation [1]. In the long-term, any remaining anthropogenic CH_4_ emissions, e.g., linked to food production, must be offset through negative emission options such as using dietary interventions (e.g., feed supplements, additives, or ingredients) to reduce GHG emissions from ruminants, improved pastures, and management systems [21]. 

### 1.3. The Role of Ruminants’ Diet in Mitigation of CH_4_ Emissions

Dietary manipulation is an attractive and effective way to mitigate CH_4_ emissions due to the direct effect of diet on rumen fermentation patterns that could lead to decreased enteric CH_4_ production [22,23,24]. In vitro and in vivo studies [25,26,27] have demonstrated that rumen fermentation measures, such as volatile fatty acids (VFA) concentration, gas/CH_4_ production, and dry matter digestibility (DMD) relate to the rumen microbial population, which in turn depends on the ruminant diet. 

A large number of studies have focused on dietary strategies to mitigate CH_4_ emissions from ruminants [15,25,28]. Dietary supplements and additives are used in livestock production to enhance feed-use efficiency, ruminant product quality, and the performance and health of the animal [26]. Recent advances in understanding methanogenesis have promoted and explored feed additives that can decrease CH_4_ emissions to varying degrees, including using dietary lipids, medium-chain fatty acids, polyunsaturated fatty acids, probiotics, plant-derived bioactive compounds, and essential oils [27,29,30,31,32]. Ionophores such as monensin have also been reported to inhibit rumen methanogenesis [33,34]. However, since the European Union (EU) banned antibiotics as feed additives in 2006 due to concerns about antimicrobial resistance in food supply chains [35], interest in using plant-based feed additives (essential oils, plant extracts, and plant-derived bioactive compounds) to reduce enteric CH_4_ emissions has increased [36].

Dietary manipulation is an attractive and effective way to mitigate ruminant-derived CH_4_ emissions due to the direct influence of feed on rumen fermentation patterns which can lead to decreased CH_4_ production. Garlic contains a number of active metabolites that could impact rumen fermentation, decreasing CH_4_ synthesis by rumen microbes and increasing propionate production in the rumen [37,38,39]. A detailed review of the literature around the potential use of garlic to decrease CH_4_ emissions is presented in Section 3 of this review.

## 2. An Introduction to Rumen CH_4_ Synthesis

### 2.1. The Rumen Microbiome and Metabolic Pathways of CH_4_ Synthesis in the Rumen

Ruminants have a unique digestive system comprised of four chambers: the reticulum, rumen, omasum, and abomasum [40,41]. The most significant among the four chambers (approx. 80% of the total volume) is the rumen, which contains a diverse and dynamic population of microorganisms that allow ruminants to break down plant material containing cellulose and hemicellulose via anaerobic fermentation [40,42]. Bacteria and protozoa account for the most significant fraction of microbial biomass (50–70%), followed by fungi (8–20%) [43,44]. These microorganisms make up a complex microbial ecosystem in the rumen, living in a symbiotic relationship with the ruminant hosts, which assists with the efficient conversion of plant biomass (rich in structural polysaccharides) into a major energy substrate i.e., VFA for the ruminant host [43,45]. For large herbivores such as dairy cows and beef cattle, this energy resource makes up 70% of the dietary energy [43].

According to Sirohi, et al. [46], rumen bacteria are the most diverse group accounting for 10^10^–10^11^ cells/mL of rumen contents: archaea, mainly methanogens, account for 10^7^–10^9^ cells/mL, fungi account for 10^3^–10^6^ cells/mL, and protozoa account for 10^4^–10^6^ cells/mL. Most of the bacteria in the rumen are strict anaerobes; they are actively involved in the breakdown of lignocellulosic feed ingredients through different enzymatic activities, which are also classified as fibrolytic, amylolytic, proteolytic, lipolytic, ureolytic, and tanniolytic bacteria [33,34,47,48]. 

To date, very few methanogenic species have been isolated from the rumen; Holotrich ciliate protozoa are highly active in the rumen and produce H_2_ that methanogens use to produce CH_4_. The interactions between bacteria and protozoa are essential and could play a critical role in the CH_4_ production pathways [44,49]. The removal of protozoa from the rumen is associated with decreased CH_4_ emission [44,50]. 

In the symbiotic relationship between the ruminant and the rumen microbial ecosystem, ruminants maintain the rumen in an anaerobic state with a stable temperature of around 39 °C and a pH ideal for microbial growth [51,52,53]. Production of CH_4_ in ruminants starts with different ruminal microorganisms, bacteria, protozoa, and fungi when they hydrolyse and ferment complex feed components such as proteins and polysaccharides into simple products, including amino acids, sugars, and alcohols [54]. 

**Figure 2:**
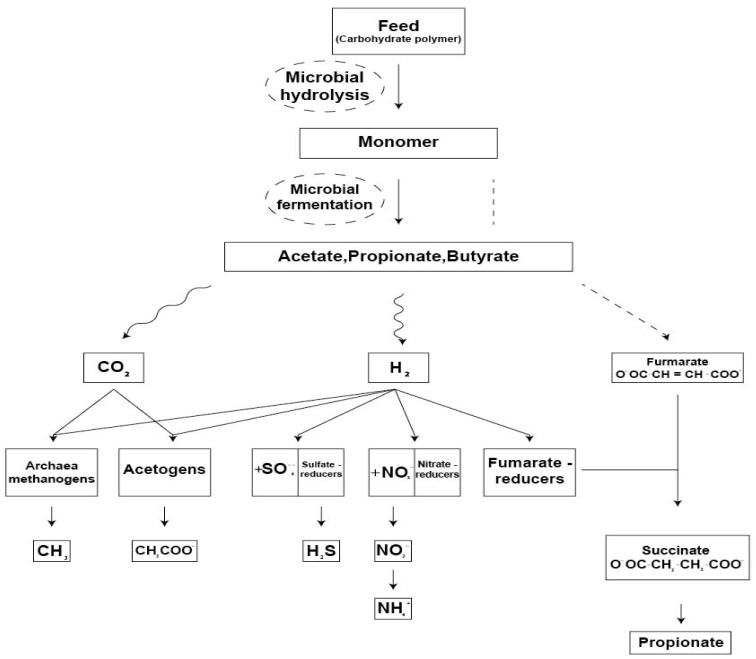
Biochemical pathways for CH_4_ synthesis (adapted from [24]). This figure is excluded from the CC BY licence under which this article is published.

The products are further fermented to VFA, H_2,_ and CO_2_ by both the primary fermenters and other microbes that cannot hydrolyse complex polymers by themselves [55]. It enables the high conversion efficiency of cellulose and hemicellulose, and CH_4_ represents a by-product of this process produced by certain microbes (methanogens) [56]. It is estimated that a cow produces 250–500 g/d CH_4_ [57]. The gaseous waste products of enteric fermentation, CO_2_, and CH_4,_ are mainly removed from the rumen by eructation [52]_._ Methane synthesis in the reticulorumen is an evolutionary adaptation that enables the rumen ecosystem to dispose of excess H_2_, which may otherwise accumulate and inhibit carbohydrate fermentation and fibre degradation [58]. Disposal of excess H_2_ produced by direct inhibition of CH_4_ production results in increased concentrations of other H_2_ sinks, such as propionate and butyrate [59]. Methanogens are at the bottom of this trophic chain and use the end products of fermentation as substrates (Figure 2).

Methanogens are anaerobic microorganisms that have three coenzymes that have not been observed in any other microorganisms, which allow them to produce CH_4_ from methyl coenzyme M [60]. It has been estimated that there are between 360–1000 species of methanogens; however, until this point, only six genera have been identified, and eight species have been cultured [53,61]. The predominant genus in the rumen is *Methanobrevibacter*, and from this genus, the most predominant species are *ruminantium*, *smithii*, and *mobile* [60]. Most methanogens grow at pH 6–8, although some species can survive in a wider range from 3–9.2 [49,62]. 

Three types of methanogenic pathways are involved in CH_4_ synthesis, namely hydrogenotrophic (reduction of CO_2_ coupled to the oxidation of H_2_), methylotrophic (conversion of methyl-group-containing compounds), and acetoclastic [63]. The hydrogenotrophic pathway is generally recognised as the main pathway to remove H_2_, through which methanogens can utilise H_2_ as an electron donor to reduce CO_2_ to CH_4_. Newly recognised methanogens use a range of methyl donor compounds and CO_2_ for CH_4_ production, suggesting that other pathways may be identified [61]. The draft genome of *Candidatus Methanomethylophilus Mx1201*, a methanogen isolated from the human gut belonging to the rumen cluster C, more recently categorised into the order *Methanomassiliicoccales* [64], contains genes for methylotrophic methanogenesis from methanol and tri-, di-, and monomethylamine [65]. In artificial systems, such as biogas production facilities, acetate is recognised as an important substrate for methanogens, which is referred to as acetoclastic methanogenesis [66]. A comprehensive understanding of the functionality of methanogens and their CH_4_-producing pathways may provide insights into effective CH_4_ abatement strategies. 

### 2.2. Targeted Manipulation of Ruminant Metabolic Pathways to Reduce CH_4_ Synthesis

Methane production in the rumen can represent a loss of up to 12% digestible energy [57] . Decreasing enteric CH_4_ emissions by ruminants without compromising animal production is desirable as a strategy both to decrease global warming effects and to improve feed conversion efficiency [16,67]. The type of feed and the presence of electron acceptors other than CO_2_ in the rumen will significantly influence the presence and activity of H_2_ producers and users [54,57]. This is because pathways other than methanogenesis can also consume H_2_ and thus potentially compete with and decrease methanogenesis in the rumen [54].

Dietary manipulation may rechannel the H_2_ produced during ruminal fermentation from CH_4_ production to propionate synthesis in the rumen [68,69]. However, the rumen ecosystem is very complex, and the ability of this system to efficiently convert complex carbohydrates to VFA is partly due to the effective removal of H_2_ by reducing CO_2_ to produce CH_4_. Thus, inhibition of methanogenesis is often short-lived, as the system’s ecology is such that it often returns to the initial level of CH_4_ production through various adaptive mechanisms [58]. Issues surrounding chemical residues, toxicity, and high cost, can also limit the utilisation of this strategy in animal production [70].

Another potential pathway is a targeted effect on certain microbial populations [31,71]. Plant-derived bioactive compounds are volatile components and aromatic lipophilic compounds which contain chemical constituents and functional groups such as terpenoids, phenolics, and phenols, which have potent antimicrobial activities. [32,72,73,74,75]. Methanogenesis decreases with the application of plant-derived bioactive compounds, primarily by reducing protozoa. Methanogenesis decreases by disrupting cell membranes due to the lipophilic nature of plant-derived bioactive compounds, decreasing protozoa and methanogens [71,76]. Therefore, the inclusion of plant-derived bioactive compounds in ruminant diets is a potential strategy to mitigate rumen CH_4_ synthesis [77]. 

A targeted approach to reducing CH_4_ emissions by dietary manipulation will therefore: (i) need to have a long-term effect by overcoming any adaptation to dietary changes, (ii) should not have a detrimental effect on the digestion of other dietary nutrients, which may occur if the rumen microbiome is altered in any way, (iii) should not have negative impact on animal health, and (iv) should not make animal-origin food products unsafe for human consumption.

## 3. Garlic and Ruminant CH_4_ Emissions

### 3.1. The Need to Exploit Plant-Derived Bioactive Compounds 

In livestock production, the use of antibiotics as growth promotors in animal feed is highly objectionable due to their residual effects and the risk of antimicrobial resistance development [78]. Garlic (*Allium sativum*) has been applied pharmaceutically since ancient times in nearly every known civilisation, has been widely used as a foodstuff in the world, and is “generally recognized as safe” (GRAS) as a food flavouring agent by the U.S. FDA, making them ideal candidates to use as feed additives in livestock production [79]. However, plant-derived bioactive compounds also exhibit antimicrobial activity and, therefore, can affect the rumen microbial ecosystem directly [36,80,81,82].

Antimicrobial properties of organosulphur compounds in garlic have shown a bactericidal effect [83,84,85,86], and hence, garlic extract and some of their compounds have been extensively investigated as a potential way to modify the rumen microbiome. Garlic is a plant that can greatly alter microbial ecosystems within the gastro-intestinal tract (GIT) of cattle [87]. Table 1 shows previously reported antimicrobial activities from garlic and its compounds (antifungal, antiprotozoal, antibacterial). The complex composition of garlic also involves a paradoxical outcome in the GIT microbiome [88]; at the same time, garlic is rich in indigestible polysaccharides, such as fructans, which act as a prebiotic for specific GIT microbiota [89].

In recent years, plant-derived bioactive compounds (e.g., organosulphur, saponins, and tannins) with diverse biological activities have been investigated for their potential as alternatives to growth-promoting antibiotics in ruminant production [72,90,91] and their potential mechanism of action as rumen modulators and inhibitors of CH_4_ production in the rumen [91,92]. To date, garlic supplementation in ruminant diets has shown a variable CH_4_ reduction in both in vitro and in vivo studies [87,93,94]; these are summarised in Table 2. 

### 3.2. Effect of Garlic on CH_4_ Emissions: In Vitro Assessments

Based on batch culture and dual flow continuous culture studies, the supplementation of garlic oil (300 mg/L) and allicin (a sulphur-containing bioactive compound in garlic; 300 mg/L) decreased CH_4_ yield (mL/g dry matter (DM)) by 73.6 and 19.5%, respectively, compared with control basal diets consisting of 50:50 forage:concentrate ratio, over 24 h [37]. The inclusion of garlic extracts at 1% of the total volume of rumen fluid containing 0.3 g of timothy grass decreased CH_4_ yield (mL/g DM) by 20% compared to control after 24 h incubation [95]. Garlic powder supplementation at 16 mg/200 mg of substrate resulted in reducing CH_4_ yield (mL/g DM) by 21% with basal diets comprising 60:40 forage:concentrate ratio over 72 h using swamp buffalo rumen fluid in batch cultures [29]. The supplementation of a combination of garlic oil at 0.25 g/L, nitrate at 5 mM, and saponin at 0.6 g/L reduced CH_4_ yield (mL/g DM) by 65% at day two and by 40% at day eighteen compared with the control basal diet consisting of 50:50 forage:concentrate ratio in batch cultures [48]. 

The effects of a combination of garlic powder and bitter orange (*Citrus aurantium*) extract (Mootral) using a semi-continuous in vitro fermentation (Rumen Simulation Technique, RUSITEC) demonstrated that the treatment effectively decreased CH_4_ yield by 96% (mL/g DM) by altering the archaeal community without exhibiting any negative effects on fermentation [96]. The study showed that a mixture of garlic and citrus extracts effectively decreased CH_4_ production in all feeding regimens without adversely affecting nutrient digestibility. Furthermore, a mixture of garlic and citrus extracts supplementation improved rumen fermentation by increasing the production of total VFA. 

The supplementation of whole garlic bulb decreased CH_4_ yield (mL/g DM) by 55% at 0.5 mL/30 mL in batch culture using rumen liquor of buffalo as inoculum without affecting the protozoa population [97]. The inclusion of garlic at the rate of 135 mg/g of substrate resulted in more than 20% inhibition in CH_4_ yield (mL/g DM), with no effect on gas production and a slight increase (2%) in in vitro DM degradability [98]; although such an inclusion rate is rather unrealistic for application at the commercial level. The effect of the inclusion of garlic oil on CH_4_ and VFA production based on in vitro is also influenced by diet and dose-dependent factors [99]. 

Some studies on ruminants have shown that garlic extracts improved nutrient use efficiency by decreasing energy loss as CH_4_ or ammonia nitrogen in continuous rumen culture [39,100,101]. Almost complete inhibition of methanogenesis has been demonstrated using garlic oil distillate without affecting feed organic matter degradation in experiments using RUSITEC [102]. These studies have consistently shown the potential of garlic supplementation in reducing CH_4_ production [48,103], while the effect on short-chain fatty acids (SCFA) production is more variable. Previous studies also observed an increase in total SCFA concentrations with moderate garlic oil concentrations [37]. Additionally, most studies reported an increase in the molar proportion of butyrate, often accompanied by a decrease in acetate proportion, whereas the effects on other SCFA and digestibility can vary [37,48,103].

Variations in the concentration and effect of individual substances in garlic extract and the type of diet can contribute to these differences [37,104]. Since different garlic varieties can vary substantially in different concentrations of compounds that affect CH_4_ emissions, the efficacy of garlic in reducing CH_4_ emissions may also depend on the variety [29,105]. However, the role of garlic and its bioactive compounds in enteric CH_4_ mitigation still remains unclear due to limited data on the mode of action related to CH_4_ mitigation potential .

### 3.3. Effect of Garlic on CH_4_ Emissions: In Vivo Assessments

Based on an in vivo study, the supplementation of a feed additive based on citrus and garlic extracts (Mootral) at 15 g/d in steers’ diets caused a decrease of 23% in CH_4_ yield after 12 weeks [106]. Steers (n = 20) receiving the Mootral treatment had lower CH_4_ production than the steers receiving the control treatment over time with no effect on DMI, average daily gain, and feed conversion efficiency. Dietary supplementation of allicin at 2 g/d for 42 d decreased CH_4_ yield (mL/g DM) by 6% compared to a control diet in sheep [107]. The inclusion of garlic extract directly affects rumen archaea, which are the microorganisms primarily responsible for CH_4_ synthesis in the rumen [37]. This hypothesis is supported by further in vivo research that reported the effect of garlic oil on the diversity of methanogenic archaea in the rumen of sheep [108]. The supplementation of garlic oil at different doses (20 g–35 g/kg DM/day) resulted in CH_4_ reduction (mmol/L of VFA) at 21.96 [109]. A decrease in CH_4_ production scaled to digested NDF intake when diallyl disulphide (DAD) was supplemented at 4 g/d in sheep [110]. The supplementation of 7% coconut oil and 100 g/d of garlic powder in buffalo diet improved the rumen ecology by increasing amylolytic and proteolytic bacteria while the protozoal population decreased by 68–75% and the CH_4_ yield (g/kg DMI) decreased by 9% without changing nutrient digestibility [111]. Other studies demonstrated no long-lasting effects on CH_4_ production when anti-methanogenic treatments (essential garlic oil and linseed oil at 3 μL/kg BW and 1.6 mL/kg BW, respectively) were given to neonatal lambs [112]. However, early-life intervention induced modifications in the composition of the rumen bacterial community of lambs that persisted after the intervention ceased with little or no effect on archaeal and protozoal communities [112]. 

Feeding garlic bulbs at the rate of 1% of DMI resulted in 11% inhibition in CH_4_ yield (g/kg DMI) in sheep (fed a diet with a 50:50 concentrate-to-roughage ratio), along with an increase in nutrient digestibility. Methane was decreased up to 31% when supplemented with garlic powder at the rate of 2% of DMI without affecting the digestibility of nutrients and milk composition compared to the control group in lactating murrah buffaloes [113]. The supplementation of freeze-dried garlic leaves (FDGL) at 2.5 g/kg DM/day of sheep diet resulted in a reduction of CH_4_ yield (g/kg DMI) by 9.7% [114]. 

Bioactive compounds derived from plants also have antimicrobial activity and, therefore, can affect the rumen microbial ecosystem. Although it might be argued that similar to the concept of developin antimicrobial resistance, there is a risk of microbes developing resistance to garlic bioactive compounds after long exposure periods. The antimicrobial properties of organosulphur compounds from garlic include a bactericidal effect. Garlic extract and some of its compounds have been studied extensively as potential means to modify the rumen microbiome. Reports on the effect of garlic on CH_4_ emissions, both in vitro and in vivo, are inconsistent between studies and applications in terms of efficient livestock production and limited ability to maintain its effects over longer periods of time. This may be due to the effect of garlic supplementation on rumen fermentation depending on the type and dosage of garlic components which vary in bioactive components, substrate composition, and composition of microbial population in the inoculum.

**Table 1:** Antifungal, antiprotozoal, antibacterial, antiviral of garlic and its compounds.

Form	Garlic Bioactive Compound (Mode of Action)	Antibacterial	Antiprotozoal	Antifungal	Reference
DAS					
DAS (purity, 97%)	Diallyl sulphide (binding to thiol-containing proteins/enzymes in bacterial cells)	*Cronobacter sakazakii*	ND	ND	[115]
Garlic extracts					
Garlic extracts	ND	ND	*Taenia taeniaeformis*, *Hymenolepis microstoma*, *H. diminuta*, *Echinostoma caproni*, and *Fasciola hepatica*	ND	[116]
Garlic extracts	Thiosulphinates and Allicin (thiol enzyme inhibition and preventing the parasite’s RNA, DNA, and protein synthesis)	ND	*Blastocystis* spp.	ND	[117]
Garlic extracts	DATS (affecting the fungal cell wall and causing irreversible ultrastructural changes in the fungal cells, leading to loss of structural integrity)	ND	ND	*Trichophyton verrucosum*, *T.mentagrophytes*, *T. rubrum*, *Botrytis cinerea*, *Candida* species, *Epidermophyton floccosum*, *Aspergillus niger*, *A. flavus*, *Rhizopus stolonifera*, *Microsporum gypseum*, *M. audouinii*, *Alternaria alternate*, *Neofabraea alba*, and *Penicillium expansum*	[118]
Garlic extracts	Allicin (oxidative interaction with important thiol-containing enzymes)	*Bacillus*, *Escherichia*, *Mycobacterium*, *Pseudomonas*, *Staphylococcus*, *and Streptococcus*	ND	*Aspergillus niger*, *Penicillium cyclopium*, *and Fusarium oxysporum*	[119]
Garlic extracts	Allicin (reacts with cysteine-containing Burkholderia enzymes involved in key biosynthetic pathways)	*B. cenocepacia* C6433	ND	ND	[120]
Garlic extracts	Allicin (interferes with RNA production and lipid synthesis)	*Bacillus subtilis*, *Staphylococcus aureus*, *Escherichia coli*, and *Klebsiella pneumonia*	ND	*Candida albicans*	[121]
Garlic extracts	Allicin (interferes with RNA production and lipid synthesis)	*S. aureus*	ND	ND	[122]
Garlic extracts	Spasmolytic effect was most likely mediated through Ca^2+^-channel inhibition	*Salmonella enteritidis*, *Escherichia coli*, *Proteus mirabilis*, and *Enterococcus faecalis*	ND	ND	[123]
Garlic extracts	Allicin (reduced serum total oxidative status, malondialdehyde, and nitric oxide production, and increased total thiols)	ND	ND	*Meyerozyma guilliermondii* and *Rhodotorula mucilaginosa*	[124]
Garlic extracts	ND	*Bacillus*, *Enterobacter*, *Enterococcus*, *Escherichia*, *Klebsiella*, *Listeria*, *Pseudomonas*, *Salmonella*, and *Staphy lococcus*	ND	*Candida albicans*	[125]
Garlic oil					
Garlic oil	DAS (the presence of the allyl group is fundamental for the antimicrobial activity of these sulphide derivatives when they are present in *Allium*)	*Staphylococcus aureus*, *Pseudomonas aeruginosa*, *and Escherichia coli*	ND	ND	[126]
Garlic oil	Ajoene (inhibiting the human glutathione reductase and *T. cruzi* trypanothione reductase)	ND	*Cochlospermum planchonii*, *Plasmodium*, *Giardia*, *Leishmania*, and *Trypanosoma.*	ND	[127]
Garlic oil	DAS (the richness in sulphur atoms may have contributed to the effectiveness of the EO activity)	*Staphylococus aureus*, *Salmonella Typhimurium*, *Listeria monocytogenes*, *Escherichia coli*, *Campylobacter jejuni*	ND	ND	[128]
Garlic oil	Allicin (inactivation of allicin by cysteine groups of mucin or other gastrointestinal bacteria)	*Campylobacter jejuni*	ND	ND	[129]

DAS: Diallyl sulphide; DATS: Diallyl Trisulphide; ND: Not Determined

**Table 2:** Effect of garlic on CH_4_ emissions based on in vitro and in vivo.

Type of Study	Garlic Form Supplementation	Level of Supply	Basal Diet	CH_4_ Yield	Reference
In Vitro					
Batch culture					
Batch culture (sheep rumen fluid)	Garlic and citrus extracts	0%, 10%, and 20% of DMI	Concentrate and grass at 50:50 ratio	↓ 11% (from 11.12 mL/g DM to 9.89 mL/g DM)	[130]
Batch culture (sheep rumen fluid)	Bulb of garlic	70 mg	450 mg DM substrate (a mixture of lucerne hay (500 g/kg), grass hay (200 g/kg), and barley (300 g/kg))	↓ 9.8% (from 1.32 mmol/g DM to 1.19 mmol/g DM)	[98]
Batch culture (sheep rumen fluid)	ALL and DAD	0.5, 5, and 10 mg/L	1:1 alfalfa hay:concentrate either (HF inoculum; 700:300 alfalfa hay:concentrate; 4 sheep) or (HC inoculum, 300:700 alfalfa hay:concentrate; 4 sheep)	ND	[39]
Batch culture (sheep rumen fluid)	Garlic oil	0, 20, 60, 180, or 540 mg/L	300 mg MC (500:500 alfalfa hay:concentrate), and the other 4 were fed HC (150:850 barley straw:concentrate)	↓ 12.1% (from 0.262 mmol/L of VFA to 0.257 mmol/L of VFA)	[99]
Batch culture (cow rumen fluid)	Garlic extracts	1% of total volume	0.3 g of timothy	↓ 20% (from 40.2 mL/g DM to 32.5 mL/g DM)	[95]
Batch culture (buffalo rumen fluid)	Coconut oil and garlic powder	0:0, 16:0, 8:4, 4:8, and 0:16 mg	200 mg DM (60:40 roughage (R) and concentrate (C) ratio were used as substrates)	ND	[29]
Batch culture (sheep rumen fluid)	Garlic oil and cinnamaldehyde	0, 20, 60, 180, and 540 mg/L	Forages and concentrates 50:50 alfalfa hay:concentrate diet (MC) and 15:85 barley straw:concentrate diet (HC)	ND	[104]
Batch culture and dual flow continuous culture (cow rumen fluid)	Garlic oil	3, 30, 300, and 3000 mg/L	50:50 forage:concentrate diet	↓ 73.6% (from 0.20 mmol/L of VFA to 0.07 mmol/L of VFA)	[37]
Batch culture (cow rumen fluid)	Combination of garlic oil, nitrate, and saponin	Garlic oil (0.25 g/L), nitrate (5 mM), and quillaja saponin (0.6 g/L)	400 mg of ground feed substrate. The feed substrate is a mixture of alfalfa hay and a dairy concentrate feed at a 50:50 ratio	↓ 65% at day 2 (from 29.1 mL/g DM to 10.3 mL/g DM) and by 40% at day 18 (from 21.4 mL/g DM to 13 mL/g DM)	[48]
Batch culture (cow rumen fluid)	Garlic powder	2–6% of DMI	Concentrate and wheat straw at a 50:50 ratio	ND	[113]
CCF					
CCF (goat rumen fluid)	PTS	200 μL/L/day	Alfalfa hay and concentrate in a 50:50 ratio	↓ 48% (from 249 mmol/L of VFA to 129 mmol/L of VFA)	[131]
Rusitec					
Rusitec (cow rumen fluid)	Mootral (garlic and citrus extract)	1–2 g	7 g hay and 3 g concentrate	↓ 96% (from 10.70 mL/g DM to 0.40 mL/g DM)	[96]
Rusitec (cow rumen fluid)	Garlic oil	300 mg/L	A basal diet (15 g DM/d) consisting of ryegrass hay, barley, and soya bean meal (1:0.7:0.3)	↓ 91% (from 7.96 mL/g DM to 0.73 mL/g DM)	[102]
In Vivo					
Buffalo					
Buffalo	Coconut oil and garlic powder	7% coconut oil plus 100 g/d of garlic powder	Rice straw ad libitum, concentrate 0.5% BW	↓ 9% (from 27.5 mmol/L of VFA to 25 mmol/L of VFA)	[111]
Buffalo	Garlic powder	2% of DMI	Concentrate and roughage diet which comprised of concentrate mixture, berseem, and wheat straw	↓ 33% (from 40.70 g/kg DMI to 27 g/kg DMI)	[113]
Buffalo	A mixture of garlic and soapnut in 2:1 ratio	2% of DMI	Wheat straw and concentrate mixture at a ratio of 60:40	↓ 12.6% (from 36.30 g/kg DMI to 31.72 g/kg DMI)	[132]
Buffalo	Mixture of garlic bulb and peppermint oil	2.5% of DMI	50% wheat straw and 50% concentrate	↓ 7.4% (from 29.17 g/kg DMI to 27.01 g/kg DMI)	[133]
Cattle					
Cattle	Mootral (garlic and citrus extract)	15 g/d	TMR at a ratio of 47% forage and 53% concentrate	↓ 23.2% (from 19.4 g/kg DMI to 14.9 g/kg DMI)	[106]
Cattle Cattle Cattle	Garlic powder A mixture of mangosteen peel, garlic, and urea pellet	40 g/d 200 g/d 200 g/d	Concentrate at 5 g/kg BW with UTRS fed ad libitum Rice straw ad libitum and concentrate were fed at 0.5% of BW Concentrate at 0.5% of BW while rice straw was fed ad libitum	↓ 5% (from 29.3 mmol/L of VFA to 27.9 mmol/L of VFA) ↓ 6.5% (from 27.6 mmol/L of VFA to 25.8 mmol/L of VFA)	[87] [134] [134] [135]
Goat					
Goat	Garlic oil	20–35 g	600 g/kg DM of concentrate and 400 g/kg DM of cowpea/maize silage in a ratio of 1:3	ND	[109]
Sheep					
Sheep	ALL	2 g/head day	TMR	↓ 7.7% (from 66.1 g/kg DMI to 61 g/kg DMI)	[107]
Sheep	FDGL	2.5 g/(kg BW0.75·d)	Mixed hay plus concentrate at 60:40 ratio	↓ 10% (from 28.05 g/kg DMI to 25.34 g/kg DMI)	[114]
Sheep	Garlic powder	0.5% concentrate (DM)	Concentrate to rice straw at ratio of 30:70	↓ 6.6% (from 42.3 g/kg DMI to 39.5 g/kg DMI)	[136]
Sheep	Combined garlic essential oil and linseed oil	Linseed oil (1.6 mL/kg BW) and garlic essential oil (3 μL/kg BW)	Free access to a natural grassland hay 921.1 g DM/kg and concentrate 889.0 g DM/kg	↓ 19.6% (from 19.68 g/kg DMI to 15.81 g/kg DMI)	[112]

BW: Body Weight; CCF: Continuous-Culture Fermenters; DAD: Diallyl Disulphide; DM: Dry Matter; DMD: Dry Matter Digestibility; DMI: Dry Matter Intake; FDGL: Freeze-Dried Garlic Leaves; HC: High Concentrate; HF: High Forage; MC: Medium Concentrate; ND: Not Determined; ALL: Allicin; PTS: Propyl Propane Thiosulphinate.

## 4. Bioactive Compounds in Garlic That Decrease CH_4_ Emissions and the Potential Effect on Biochemical Pathways

**Figure 3:**
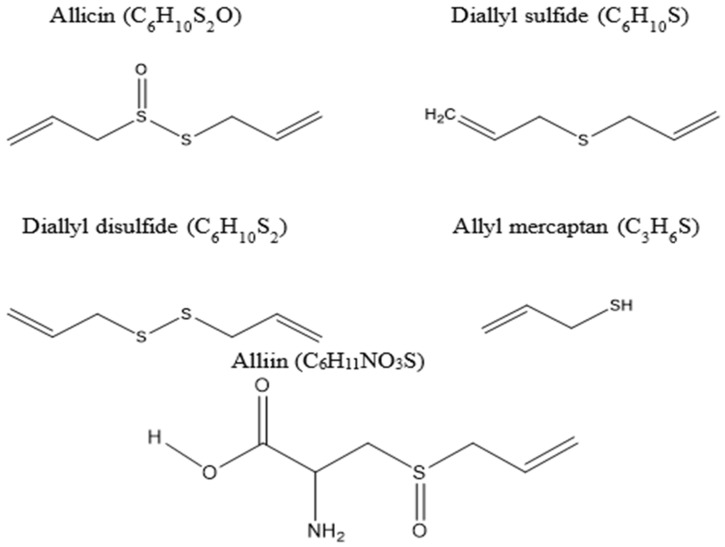
Chemical structures of allicin (C_6_H_10_S_2_O), diallyl sulphide (C_6_H_10_S), diallyl disulphide (C_6_H_10_S_2_), allyl mercaptan (C_3_H_6_S), and alliin (C_6_H_11_NO_3_S).

Garlic contains the organosulphur compounds allicin (C_6_H_10_S_2_O), alliin (C_6_H_11_NO_3_S), diallyl sulphide (C_6_H_10_S), diallyl disulphide (C_6_H_10_S_2_), and allyl mercaptan (C_3_H_6_S) [137,138,139,140] (Figure 3). These compounds are widely known for their unique therapeutic properties and health benefits, as they act as antioxidants to scavenge free radicals [141]. Garlic-derived organosulphur compounds demonstrate different biochemical pathways that may provoke multiple inhibitions [142]. One potential pathway for the direct inhibition of methanogenesis by garlic is via the inhibition of CH_4_-producing microorganisms such as archaea [142]. Archaea possess unique glycerol-containing membrane lipids linked to long-chain isoprenoid alcohols, which are essential for cell membrane stability. The synthesis of isoprenoid units in methanogenic archaea is catalysed by the enzyme hydroxyl methyl glutaryl coenzyme A (HMG-CoA) reductase. Garlic oil is a potent inhibitor of HMG-CoA reductase Gebhardt and Beck [142]; as a result, the synthesis of isoprenoid units is inhibited, the membrane becomes unstable, and cells die. The effect of garlic bioactive compounds in ruminants has been reported in Table 3.

Diallyl sulphide (DAS) has shown small effects on rumen microbial fermentation [37]. It has been suggested in various studies that the antimicrobial potency of allyl sulphides in garlic oil increases with each additional S atom [143,144]. This could explain why supplementation of DAD (which contains two S atoms) resulted in more potent effects compared with diallyl sulphide (DAS) (containing one S atom). Supplementation of DAD at 80 μL/L/day and propyl propane thiosulphinate (PTS) at 200 μL/L/day strongly inhibited CH_4_ yield (g/kg DMI) by 62% and 96%, respectively, in batch cultures after 24 h incubation of the ruminal fluid of goats [131]. 

Supplementation of allicin at 2 g/head/day effectively enhanced OM, N, NDF, and ADF digestibility and decreased daily CH_4_ yield (g/kg DMI) in ewes, probably by decreasing the population of ruminal protozoa and methanogens [107]. Supplementary allicin can also decrease the ruminal concentration of ammonia by 14% but can increase the total VFA produced by up to 14.3% [100,107,110]. Significant increases in the populations of *F. succinogenes*, *R. flavefaciens*, and *B. fibrisolvens* in ewes supplemented with allicin have also been observed [135]. It is well established that CH_4_ production has been positively correlated with more acetate production and negatively correlated with increased propionate production [145] because propionate synthesis is a main pathway for H_2_ consumption, representing a competitive and alternative pathway to methanogenesis [70,146]. Allicin has been found to alter rumen VFA production so that less acetate and more propionate and butyrate are produced, and this may be due to an abundance of the *Prevotellaceae* and *Veillonellaceae* families [112]. *Prevotellaceae* is one of the predominant families in rumen fluid, and it is well known to produce propionate by utilising H_2_ produced during carbohydrate fermentation [147]. 

Dietary garlic constituents are transformed into various metabolites in a biological system. Busquet, Calsamiglia, Ferret, Carro and Kamel [37] observed that allyl mercaptan is a common metabolite of allium-derived compounds as obtained after incubation of allicin and other allyl sulphides in fresh blood at 37 °C or gastric fluids [137]. Diallyl disulphide and allyl mercaptan resulted in a less potent effect than garlic oil in increasing in vitro rumen fermentation and decreasing CH_4_ production, suggesting a possible synergistic effect between the different compounds present in the garlic oil [37]. In the specific case of garlic oil, the CH_4_ mitigating effect may be directly attributed to the toxicity of organosulphur compounds, such as diallyl sulphide and allicin, to the methanogens [148].

Garlic extracts have demonstrated effectively decreased CH_4_ production and improved rumen fermentation by increasing the production of total VFA at 200 g/kg of the feed [130]. Supplementation with garlic extracts has been associated with a lower abundance of the family *Methanobacteriaceae*, the major CH_4_ producer in the rumen [96]. This was connected to the toxicity of the organosulphur compounds of garlic, such as diallyl sulphide and allicin, in inhibiting certain sulphydryl-containing enzymes essential for the metabolic activities of methanogenic archaea [48]. This interaction has been demonstrated by the loss of activity of some thiol-containing enzymes (e.g., papain and alcohol dehydrogenases) and by the reaction between different organosulphur compounds and cysteine to form other substances by a thiol-disulphide exchange reaction [143].

The constituents of dietary garlic are converted into various metabolites in biological systems, which can cause synergistic effects between different compounds in garlic. It can therefore cause different forms of garlic to have different bioactive components. This compound can potentially impact CH_4_ reduction, which is directly related to the toxicity of organosulphur compounds to methanogens. 

**Table 3:** The effect of bioactive compounds in ruminants.

Animal	Basal Diet	Garlic form Supplementation	Bioactive Compound	Level of Supply	Effects	Reference
Buffalo						
Buffalo	Concentrate was offered at 0.5% of BW^,^ while rice straw was given on ad libitum basis	Coconut oil and garlic powder	ND	7% coconut oil plus 100 g/d of garlic powder	↑ BUN^22^; C_3_; Total bacteria population; Amylolytic and proteolytic bacteria; rumen ecology ↓ CH_4_; Total VFA; C_2_; C_2_/C_3_ ratio; protozoal population	[111]
Buffalo	Concentrate and roughage diet which comprised of concentrate mixture, berseem, and wheat straw	Garlic powder	ND	2% of DMI	↑ Milk production; Digestibility↓ CH_4_	[113]
Buffalo Buffalo	Wheat straw and concentrate mixture at a ratio of 60:40 50% wheat straw and 50% concentrate mixture	A mixture of (garlic and soapnut in 2:1 ratio A mixture of garlic bulb and peppermint oil	ND ND	2% of DMI 2.5% of DMI	↑ urinary nitrogen; feed conversion efficiency ↓ CH_4_; faecal nitrogen ↓ CH_4_	[132] [133]
Cattle						
Cattle	TMR according to the National Academies of Sciences, Engineering, and Medicine	Mootral (garlic and citrus extract)	ALL and flavonoid	15 g/d	↓ CH_4_ CO_2_ and O_2_ did not differ between treatments	[106]
					DMI, average daily gain, and feed efficiency remained similar in control and supplemented steers	
Cattle Cattle	Concentrate at 5 g/kg BW UTRS fed ad libitum Rice straw ad libitum and concentrate were fed at 0.5% of BW	Garlic powder	ALL, ajoene, S-allylcysteine, DAD, S-methylcysteine sulphoxide, and S-allylcysteine A mixture of mangosteen peel, garlic, and urea pellet	40 g/d 200 g/d	↑ pH; C_3_; rumen fermentation efficiency ↓ CP digestibility; NH_3_-N; C_2_; CH_4_; Population sizes of bacteria and protozoa; proteolytic bacteria; amylolytic and cellulolytic bacteria ↑ NH_3_-N; C_3_; bacterial population; rumen fermentation, microbial protein synthesis ↓ CH_4_; protozoa population	[87] [134]
Cow						
Cow	TMR	Garlic essential oil	ALL	5 g/kg DM	↑ Feed digestibility ↓ The flow of bypass protein to the small intestine	[149]
Cow	TMR	DAD	DAD	DAD was fed at levels of 56 mg/kg DM and 200 mg/kg DM in Exp. 1 and Exp. 2, respectively. This is equivalent to 1.0 or 3.3 g/cow per day		[150]
Cow	Fed with ad libitum with UTRS and concentrate at 0.5 g kg^−1^ body weight (BW) twice daily	Garlic powder	ND	80 g d^−1^	↑ C_3_; N retention and absorption ↓ C_2_/C_3_; Protozoa	[151]
Goat						
Goat	600 g/kg DM of concentrate and 400 g/kg DM of cowpea/maize silage in a ratio of 1:3, respectively	Garlic oil	ND	20–35 g	↑ ADF & lignin digestibility, total VFA, FCR, NH_3_-N, digestibility ↓ CH_4_; Protozoa	[109]
Goat	Grass hay (Leymus chinensis, 0.38 kg/d dry matter (DM)) and concentrate (0.22 kg/d DM)	Garlic oil	ND	0.8 g/d		[152]
Sheep						
Ewe	TMR	ALL	ALL	2 g/d	OM; N; NDF; ADF digestibility ↓ CH_4_; protozoa and methanogens	[107]
Ewe	TMR based on barley-based diet	Garlic oil	ALM (26%), allyl trisulphide (18%), ALL (1.5%)	0.02 g/kg DM	↑ *Methanosphaera stadtmanae*, *Methanobrevibacter smithii* Alter the diversity of rumen methanogens without affecting the methanogenic capacity of the rumen	[108]
Lamb	A barley-based concentrate diet ad libitum	Garlic essential oil	ND	200 mg/kg DM	No effects on intake and ruminal fermentation characteristics compared to lambs fed unsupplemented diet The addition of garlic did not affect carcass characteristics or meat quality and had small effects on FA composition of back fat and liver It seems unlikely that these minor changes will have any impact on the health properties of lamb meat	[103]
Lamb	Free access to a natural grassland hay [921.1 g dry matter (DM)/kg and concentrate (889.0 g DM/kg)]	Combined garlic essential oil and linseed oil	ND	Linseed oil (1.6 mL/kg BW) and garlic essential oil (3 μL/kg BW)	↓ CH_4_; VFA A long-term early-life intervention induced modifications in the composition of the rumen bacterial community There was no persistency of the early-life intervention on methanogenesis	[112]
Lamb	According to Ministry of Agriculture of P. R. China, 2004	Garlic skin	ND	80 g/kg DM	↑ ADG; VFA; *Prevotella*, *Bulleidia*, *Howardella*, *Methanosphaera* ↓ Fretibacterium Favourably regulated pyrimidine metabolism, purine metabolism, vitamin B6 and B1 metabolism High correlations between uctuant rumen microbiota and metabolites	[91]
Sheep	Control diet (basal total mixed ration with no additive = CTR)	Raw garlic or garlic oil	ND	Dose of raw garlic (75 versus 100 g/kg DM) and garlic oil (500 versus 750 mg/kg DM)	C_3_; C_2_/C_3_ ratio ↓ NDF; ADF by garlic oil supplementation; Protozoa in a dose-independent manner; NH_3_	[105]
Sheep	Mixed hay (Hay-diet, as control) and hay plus garlic stem and leaf silage diet (GS-diet, at ratio of 9:1)	Garlic stem and leaf silage	ND	66 g/kg BW 0.75/d DM	↑ Nitrogen digestibility; C_3_; C_5_; Glucose; plasma LeuTR and WBPS NEFA	[101]
Sheep	Meadow hay (3rd cut, vented) and concentrate (barley grain and soybean meal; 700:300) offered in a 1:1 ratio	Garlic oil	DAD	5 g garlic oil or 2 g DAD/kg DM	↑ digestibility and energy use efficiency ↓ concentrate intake; Low palatability	[110]
Sheep	Mixed hay plus concentrate at 60:40 ratio	FDGL	ALL	2.5 g/(kg BW 0.75·d)	↑ NH_3_-N; Glucose ↓ CH_4_; DM ingested	[114]
Sheep	Forage to concentrate ratio of 1:1	Bulb of garlic	ND	1% of DM	↑ Nutrient digestibility (DM, OM, NDF, ADF^,^ and cellulose)	[93]
Ram	Concentrate to rice straw was 30:70 (as-fed basis)	Garlic powder	ND	0.5% concentrate (DM)	↓ CH_4_; Serum glutamic oxaloacetic transaminase	[136]

ADF: Acid Detergent Fibre; ADG: Average Daily Gain; ALL: Allicin; ALM: Allyl Mercaptan; BUN: Blood Urea Nitrogen; BW: Body Weight; C_2_: Acetate; C_3_: Propionate; C_5_: Butyrate; CP: Crude Protein; DAD: Diallyl Disulphide; DM: Dry Matter; DMI: Dry Matter Intake; FA: Fatty Acid; FCR: Feed Conversion Ratio; FDGL: Freeze-Dried Garlic Leaves; ND: Not Determined; NDF: Neutral Detergent Fibre; NEFA: Plasma Non-Esterified Fatty Acids; OM: Organic Matter; TMR: Total Mix Ratio; UTRS: Urea-Treated Rice Straw; VFA: Volatile Fatty Acid.

## 5. Nutritive Value of Garlic in Ruminants

### 5.1. Chemical Composition of Garlic

Garlic contains volatile oils and protein, comprising 1–3.6 g/kg and 160–170 g/kg, respectively [137]. In addition, it is a rich source of sulphur, potassium, phosphorus, magnesium, sodium, and calcium [119]. The sulphur content in garlic varies from 5 to 37 g/kg of DM [119]. Garlic products can be classified into garlic essential oils, garlic oil macerate, garlic powder, and garlic extract [153].

### 5.2. Effects of Garlic on Rumen Fermentation

Garlic powder and garlic oil exhibit activities on modifying rumen fermentation parameters, improving nutrient digestibility, decreasing rumen protozoa numbers, and decreasing CH_4_ emissions, and the effect of garlic extracts on the rumen microbiome have been comprehensively investigated [149,151]. The latest findings on the effect of garlic on ruminant animal productivity are summarised for both in vitro (Table 4) and in vivo determinations (Table 5).

Supplementation of garlic oil at 0.8 g/d did not greatly affect ruminal fermentation parameters (total VFA concentration and individual VFA molar proportions) but increased ammonia and microbial crude protein [152]. In addition, garlic oil altered rumen fatty acid profile by increasing the concentration of certain fatty acids e.g., t11-18:1 (TVA) and c9, t11-CLA. This appeared to be achieved as a consequence of inhibition of the final step of biohydrogenation, which can lead to the accumulation of TVA in the rumen [152]. Garlic powder supplementation at 80 g/d in steers could enhance ruminal propionate production and reduce the acetate/propionate (C_2_:C_3_) ratio by 10%, decreasing protozoa population while increasing N retention and absorption in ruminants [91]. Similarly, Ahmed, Yano, Fujimori, Kand, Hanada, Nishida and Fukuma [130] showed similar finding in in vitro studies; the supplementation of garlic and citrus extract at 20% of the substrate could improve the production of total VFA and propionate and reduce C_2_:C_3_ ratio by 27%. 

The effect of garlic oil and other organosulphur compounds (diallyl disulphide and allyl mercaptan) on rumen microbial fermentation in batch culture have been reported as resulting in lower molar proportions of acetate and higher proportions of propionate and butyrate upon supplementation of diallyl disulphide (DAD) (30 and 300 mg L^−1^ culture fluid) and allyl mercaptan (300 mg L^−1^ culture fluid) [37]. Moreover, there was a decrease in CH_4_ yield (mL/g DM) of 73.6, 68.5, and 19.5% upon administration of garlic oil, DAD, and allyl mercaptan at 300 mg/L, respectively, which may help to improve the efficiency of energy use in rumen fermentation [37]. The effects of cinnamaldehyde and garlic oil have been investigated on rumen fermentation in a dual-flow continuous culture [154]. They reported that the inclusion of garlic oil at 312 mg/L increased the small peptide plus amino acid N concentration and the proportion of propionate and butyrate and decreased the proportion of acetate and branch-chained VFA, which indicate that garlic oil affected the fermentation profile and can be used as modulators of rumen microbial fermentation [37]. However, in the experiment of Kamel, Greathead, Tejido, Ranilla and Carro [39], three levels of DAD (0.5, 5, and 10 mg/L) were investigated, but none of the treatments had a suppressing effect on CH_4_ production. Furthermore, DAD supplementation at 56 and 200 mg/kg DM levels failed to decrease CH_4_ production in vivo [150]. Other studies reported that DAD supplementation in sheep diet only tended to decrease CH_4_ yield relative to OM digested and that its potential to reduce CH_4_ production in sheep was low; despite that, it improved digestibility and energy use efficiency by promoting the growth of anaerobic rumen fungi which might increase fibre digestion [110].

Reports of garlic’s effect on rumen fermentation are inconsistent between studies. This might be the effect of various factors, such as the dose administered, the composition of the substrate, and the composition of the microbial population in the inoculum [99]. Garlic oil and garlic powder tested at high doses showed the highest impact in reducing CH_4_ emission. However, the dose level needs to be considered on how much it can be fed at the farm level. 

### 5.3. Effects of Garlic on Rumen Microbiota

Garlic has been found to modify the microbial population profile in continuous culture experiments, reducing specifically the *Provotella* spp. (mainly *P.ruminantium* and *P. briyantii*) while other microbial populations remain unaffected [92,155]. *Provotella* spp. is mainly responsible for protein degradation and amino acid deamination, suggesting that garlic oil may also affect protein metabolism in which dehydrogenase activity is required to suppress deamination when using CH_4_ inhibitors [156]. 

Endo and ectosymbiotic methanogens of protozoa can contribute around 25% of CH_4_ emission from sheep rumen fluid, but the effect of garlic by-products on protozoa numbers was highly variable between different studies [49,143]. The effect of garlic powder supplementation at 4 mg/200 mg DM in vitro fermentation systems has shown a decrease in protozoa population by 60% [29]. Supplementing a basal diet with raw garlic or garlic oil at 500 mg/kg DM decreased the number of rumen protozoa in sheep by 35% [105]. Most studies that investigated the effect of garlic components on the population of methanogens were carried out in vitro. The inclusion of garlic oil at 100 and 250 mg/L decreased methanogenic bacterial activity by 68.5 and 69%, respectively (Chaves, He, Yang, Hristov, McAllister and Benchaar [103]). Supplementation of garlic oil at 1 g/L effectively reduced the in vitro abundance of *F. succinogenes*, *R. flavefaciens*, and *R. albus* without affecting total bacteria and could reduce the abundance of archaea and protozoa population by 16.5 and 8%, respectively (Patra and Yu [32]). In addition, the increase in the population of those three cellulolytic bacteria (*F. succinogenes*, *R. flavefaciens*, and *R. albus)* could be more probably explained by the reduced populations of the protozoa that engulf bacteria [32].

Observations of the reduction of methanogens coincide with those of in vitro results. In addition, the decreased population of protozoa could also be responsible for the reduction in methanogens, as the total methanogen population declined in absolute number as well as in proportion to the total bacterial population in the absence of protozoa [157]. Garlic powder supplementation at 80 g/d did not affect the amylolytic or cellulolytic bacteria population but decreased the protozoa population by 41% (Wanapat, Khejornsart, Pakdee and Wanapat [151]). Supplementation of plant extracts (mixture of garlic and citrus extract) at 10% and 20% of the substrate reduced *Methanobacteriaceae*, which is the major CH_4_ producer in the rumen, by 94.07 and 92.70, respectively (Ahmed, Yano, Fujimori, Kand, Hanada, Nishida and Fukuma [130]). Furthermore, 20% PE effectively increased the abundance of H_2_-consuming groups such as *Prevotellaceae* and *Veillonellaceae* and reduced some H_2_-producing bacteria.

Garlic showed positive effects on rumen fermentation, improving nutrient digestibility and altering the rumen microbiome by decreasing the number of protozoa and decreasing CH_4_ emissions. However, the effects are inconsistent between studies. In addition, future research should aim to understand the mode of action of garlic and its bioactive compounds in regard to enteric CH_4_ mitigation.

## 6. Conclusions and Future Perspectives

Significant amounts of research have been conducted to identify strategies to reduce entric CH_4_ emissions, as this is a major contributor to global warming. Understanding rumen function and dynamics have been found to be important in determining dietary strategies to mitigate CH_4_ production in the rumen. Interactions between bacteria and protozoa are crucial and play a critical role in ruminal CH_4_ production pathways. The main target of dietary manipulation is either via direct inhibition of methanogens, or by altering metabolic pathways leading to the reduction of substrates for methanogenesis. Garlic and its bioactive compounds, such as allicin (C_6_H_10_S_2_O), diallyl sulphide (C_6_H_10_S), diallyl disulphide (C_6_H_10_S_2_), and allyl mercaptan (C_3_H_6_S), have demonstrated inconsistent effects in decreasing CH_4_ production during rumen fermentation. This may be due to various reasons: firstly, different types of garlic contain different amounts of bioactive compounds. Secondly, the composition of the basal diet can affect the action of garlic-origin bioactive compounds by modulating rumen metabolism. However, generally increasing the dietary dose of garlic and/or its bioactive compounds results in a decrease in CH_4_ production. Further research is needed to understand how organosulphur compounds in garlic influence methanogens and their metabolic pathways, providing insight into effective CH_4_ mitigation strategies. Generally, there will not be a single “silver bullet” for agricultural GHG emissions. Rather, this approach will have a shorter-term impact but could be combined with other dietary strategies to prevent adverse effects on rumen digestibility and fermentation. There are real opportunities for the feed industry to develop garlic-based feed additives to reduce CH_4_ emission from ruminant production. Given the far-reaching consequences of rumen fermentation on ruminant nutrition, food production, and the environment, it is not surprising that many studies have been undertaken to understand microbial populations in the rumen and ultimately manipulate them to maximise productivity while reducing the environmental impact of ruminant production.

## Figures and Tables

**Table 4:** In vitro trials that studied the effect of garlic on ruminant productivity.

In Vitro Studies	Basal Diet (Forage and Concentrate Ratio)	Garlic Form	Level of Supply	Effects	Reference
Batch culture					
Batch culture	1000 g grass/kg ration + 0 g concentrate/kg ration (100:0), 80:20, 60:40, 40:60, and 20:80	Mixture of garlic and citrus extracts	200 g/kg of the feed	↑ Gas and CO_2_; NH_3_-N_;_ Total VFA:C_3_ and C_5_ ; pH; C_2_ Did not interfere with OM and fibre digestibility Altering rumen fermentation	[130]
Batch culture	0.5 g DM of a 10:90 forage:concentrate	Garlic extract	0, 0.3, 3, 30, and 300 mg/L	↓ C_2_/C_3_ ratio; pH; C_3_ ↓ Total VFA; NH_3_-N; C_2_	[100]
Batch culture	Grass and concentrate mixture (50:50)	*Sapindus rarak* extract with or without garlic extract	1.8 g/kg *Sapindus rarak* extract + 0.25 ppm garlic extract	↑ C_3_; ruminal fermentation based on feed digestibility, fermentation products, and rumen bacterial population ↓ Crude digestibility; C_2_; Protozoa	[94]
Batch culture	450 mg DM substrate (a mixture of lucerne hay (500 g/kg), grass hay (200 g/kg), and barley (300 g/kg))	Bulb of garlic	70 mg	DM digestibility ↓ CH_4_; C_2_/C_3_	[98]
Batch culture	1:1 alfalfa hay:concentrate either (HF inoculum; 700:300 alfalfa hay: concentrate; 4 sheep) or (HC inoculum, 300:700 alfalfa hay:concentrate; 4 sheep)	ALL and DAD	0.5, 5, and 10 mg/L	↑ C_2_/C_3_ ratio at HC ↓ pH; CH_4_/VFA	[39]
Batch culture	300 mg (MC; 500:500 alfalfa hay:concentrate), and the other 4 were fed (HC; 150:850 barley straw:concentrate)	Garlic oil	0, 20, 60, 180, or 540 mg/L	C_2_/C_3_ ratio; C_5_ by garlic oil at 60, 180, and 540 mg/L with diet MC ↓ Total VFA by garlic oil 540 for MC diet; C_2_ by increasing doses of garlic oil; CH_4_	[99]
Batch culture	0.3 g of timothy	Garlic extracts	1% of total volume	↑ Total VFA; fibrolytic bacteria; *F. succinogens* C_2_/C_3_ ratio; ciliate-associated methanogen; *R. flavefaciens*	[95]
Batch culture	200 mg DM (60:40 roughage (R) and concentrate (C) ratio were used as substrates)	Coconut oil and garlic powder	0:0, 16:0, 8:4, 4:8, and 0:16 mg	↑ C_3_; *Ruminococcus albus* at 8:4 mg; at 8:4 and 0:16 mg could improve ruminal fluid fermentation in terms of VFA profile ↓ Gas production; NH_3_-N; Total VFA; C_2_/C_3_ ratio; CH_4_; Protozoa	[29]
Batch culture	Forages and concentrates 50: 50 alfalfa hay:concentrate diet (MC), and the other four received a 15:85 barley straw:concentrate diet (HC)	Garlic oil and cinnamaldehyde	0, 20, 60, 180, and 540 mg/L	↑ VFA ↓ CH_4_/ VFA ratio the effectiveness of garlic oil and cinnamaldehyde in manipulating ruminal fermentation may depend on the characteristics of the diet fed to the animals, which highlights the importance of testing these additives with different diet types	[104]
Batch culture and dual flow continuous culture	50:50 forage:concentrate diet	Garlic oil	3, 30, 300, and 3000 mg/L	Batch culture ↑ C_3_; C_5_ with supplementation of Garlic oil (30 and 300 mg/L), DAD (30 and 300 mg/L), and ALM (300 mg/L) C_2_ with supplementation of Garlic oil (30 and 300 mg/L), DAD (30 and 300 mg/L), and ALM (300 mg/L) Dual flow Continuous Culture: ↑ Efficiency of energy use in the rumen ↓ CH_4_	[37]
Batch culture	200 mg substrate	Bulb of garlic	30 mg	↑ Gas production ↓ CH_4_ Inhibited methanogenesis without adversely affecting other rumen characteristics	[97]
Batch culture	400 mg of ground feed substrate. The feed substrate is a mixture of alfalfa hay and a dairy concentrate feed at a 50:50 ratio	Combination of garlic oil, nitrate, and saponin	garlic oil (0.25 g/L), nitrate (5 mM), and quillaja saponin (0.6 g/L)	↑ NH_3_-N by nitrate at days 10 and 18 ↓ CH_4_; Feed digestion by the combinations (binary and ternary) of garlic oil with the other inhibitors at days 10 and 18; NH_3_-N by saponin, alone or in combinations, and garlic oil alone at day 2; Total VFA by garlic oil alone or garlic oil-saponin combination; Methanogens	[48]
Batch culture	Concentrate and wheat straw at a 50:50 ratio	Garlic powder	2–6% of DMI	↓ CH_4_; C_3_; C_5_	[113]
CCF					
CCF	Alfalfa hay and concentrate in a 50:50 ratio	PTS	200 μL/L/day	↑ *Prevotella; Methanobrevibacter* and *Methanosphaera* ↓ CH_4_; methanogenic archaea; Methanomicrobiales	[131]
CCF	50:50 alfalfa hay:concentrate	Garlic oil	312 mg/L	C_3_; C_5_; Small peptide; NH_3_-N ↓ C_2_; VFA	[37]
Rusitec					
Rusitec	7 g hay and 3 g concentrate	Mootral (garlic and citrus extract)	1–2 g	SCFA; C_5_ ↓ CH_4_; *Methanobacteriacea*	[96]
Rusitec	A basal diet (15 g DM/d) consisting of ryegrass hay, barley, and soya bean meal (1:0·7:0·3)	Garlic oil	300 mg/L	↑ Bacterial population ↓ CH_4_; Protozoa; NDF	[102]

ALL: Allicin; ALM: Allyl Mercaptan; C_2_: Acetate; C_3_: Propionate; C_5_: Butyrate; CCF: Continuous-Culture Fermenters; DAD: Diallyl Disulphide; DM: Dry Matter; DMI: Dry Matter Intake; HC: High-Concentrate; HF: High Forage; MC: Medium Concentrate; NDF: Neutral Detergent Fibre; OM: Organic Matter.PTS: Propyl Propane Thiosulphinate; Rusitec: Rumen Simulation Technique; SCFA: Short Chain Fatty Acid; VFA: Volatile Fatty Acid.

**Table 5:** In vivo trials that studied the effect of garlic on ruminant productivity.

In Vivo Studies	Basal Diet (Forage and Concentrate Ratio)	Garlic Form Supplementation	Level of Supply	Effects in Ruminant Productivity	References
Buffalo					
Buffalo	Concentrate was offered at 0.5% of BW^,^ while rice straw was given on ad libitum basis	Coconut oil and garlic powder	7% coconut oil plus 100 g/d of garlic powder	↑ BUN; C_3_; Total bacteria population; Amylolytic and proteolytic bacteria; rumen ecology ↓ CH_4_; Total VFA; C_2_; C_2_/C_3_ ratio; protozoal population	[111]
Buffalo	Concentrate and roughage diet which comprised of concentrate mixture, berseem, and wheat straw	Garlic powder	2% of DMI	↑ Milk production; Digestibility ↓ CH_4_	[113]
Cattle					
Cattle	TMR according to the National Academies of Sciences, Engineering, and Medicine	Mootral (garlic and citrus extract)	15 g/d	↓ CH_4_ CO_2_ and O_2_ did not differ between treatments DMI, average daily gain, and feed efficiency remained similar in control and supplemented steers	[106]
Cattle	Concentrate at 5 g/kg BW with UTRS fed ad libitum	Garlic powder	40 g/d	↑ pH; C_3_; rumen fermentation efficiency ↓ CP digestibility; NH_3_-N; C_2_; CH_4_; Population sizes of bacteria and protozoa; proteolytic bacteria; amylolytic and cellulolytic bacteria	[87]
Cow					
Cow	TMR	DAD	DAD was fed at levels of 56 mg/kg DM and 200 mg/kg DM in Exp. 1 and Exp. 2, respectively. This is equivalent to 1.0 or 3.3 g/cow per day		[150]
Cow	Fed with ad libitum with urea-treated rice straw and concentrate at 0.5 g kg^−1^ body weight (BW) twice daily	Garlic powder	80 g d^−1^	C_3_; N retention and absorption C_2_/C_3_; Protozoa	[151]
Cow	TMR	Garlic essential oil	5 g/kg DM	↑ Feed digestibility ↓ The flow of bypass protein to the small intestine	[149]
**Goat**					
Goat	600 g/kg DM of concentrate and 400 g/kg DM of cowpea/maize silage in a ratio of 1:3, respectively	Garlic oil	20–35 g	↑ ADF & lignin digestibility, total VFA, FCR, NH_3_-N, digestibility ↓ CH_4_, protozoa	[109]
Goat	Grass hay (*Leymus chinensis*, 0.38 kg/d DM) and concentrate (0.22 kg/d DM)	Garlic oil	0.8 g/d		[152]
Sheep					
Ewe	TMR based on barley-based diet	Garlic oil	0.02 g/kg DM	↑ *Methanosphaera stadtmanae*, *Methanobrevibacter smithii* Alter the diversity of rumen methanogens without affecting the methanogenic capacity of the rumen	[108]
Ewe	TMR	ALL	2 g/head day	OM; N; NDF; ADF digestibility ↓ CH4; protozoa and methanogens	[107]
Lamb	A barley-based concentrate diet ad libitum	Garlic essential oil	200 mg/kg DM	No effects on intake and ruminal fermentation characteristics compared to lambs fed unsupplemented diet The addition of garlic did not affect carcass characteristics or meat quality and had small effects on FA composition of back fat and liver It seems unlikely that these minor changes will have any impact on the health properties of lamb meat	[103]
Lamb	Free access to a natural grassland hay [921.1 g dry matter (DM)/kg and concentrate (889.0 g DM/kg)]	Combined garlic essential oil and linseed oil	Linseed oil (1.6 mL/kg BW) and garlic essential oil (3 μL/kg BW)	↓ CH_4;_ VFA A long-term early-life intervention induced modifications in the composition of the rumen bacterial community There was no persistency of the early-life intervention on methanogenesis	[112]
Lamb	According to Ministry of Agriculture of P. R. China, 2004	Garlic skin	80 g/kg DM	ADG; VFA; *Prevotella*, *Bulleidia*, *Howardella*, *Methanosphaera* ↓ *Fretibacterium* Favourably regulated pyrimidine metabolism, purine metabolism, vitamin B_6_ and B_1_ metabolism High correlations between uctuant rumen microbiota and metabolites	[91]
Sheep					
Sheep	Control diet (basal total mixed ration with no additive = CTR)	Raw garlic or garlic oil	Dose of raw garlic (75 versus 100 g/kg DM) and garlic oil (500 versus 750 mg/kg DM)	C_3_; C_2_/C_3_ ratio NDF; ADF by garlic oil supplementation; Protozoa in a dose-independent manner; NH_3_	[105]
Sheep	Mixed hay (Hay-diet, as control) and hay plus garlic stem and leaf silage diet (GS-diet, at ratio of 9:1)	Garlic stem and leaf silage	66 g/kg BW 0.75/d DM	↑ Nitrogen digestibility; C_3_; C_5_; Glucose; plasma LeuTR and WBPS ↓ Plasma non-esterified fatty acids (NEFA)	[101]
Sheep	Meadow hay (3rd cut, vented) and concentrate (barley grain and soybean meal; 700:300) offered in a 1:1 ratio	Garlic oil	5 g garlic oil or 2 g DAD/kg dietary DM	↑ Digestibility and energy use efficiency ↓ Concentrate intake; Low palatability	[110]
Sheep	Mixed hay plus concentrate at 60:40 ratio	FDGL	2.5 g/(kg BW 0.75·d)	↑ NH_3_-N; Glucose ↓ CH_4_; DM ingested	[114]
Sheep	Forage to concentrate ratio of 1:1	Bulb of garlic	1% of DM	↑ Nutrient digestibility (DM, OM, NDF, ADF, and cellulose)	[93]

ADF: Acid Detergent Fibre; ADG: Average Daily Gain; ALL: Allicin; BUN: Blood Urea Nitrogen; BW: Body Weight; C_2_: Acetate; C_3_: Propionate; C_5_: Butyrate; CP: Crude Protein; DAD: Diallyl Disulphide; DM: Dry Matter; DMI: Dry Matter Intake; FA: Fatty Acid; FCR: Feed Conversion Ratio; FDGL: Freeze-Dried Garlic Leaves; NDF: Neutral Detergent Fibre; NEFA: Plasma Non-Esterified Fatty Acids.OM: Organic Matter; TMR: Total Mix Ratio; UTRS: Urea-Treated Rice Straw; VFA: Volatile Fatty Acid.

## Data Availability

Not applicable.
